# Infections, Hospitalizations, and Deaths Among US Nursing Home Residents With vs Without a SARS-CoV-2 Vaccine Booster

**DOI:** 10.1001/jamanetworkopen.2022.45417

**Published:** 2022-12-01

**Authors:** Kevin W. McConeghy, Barbara Bardenheier, Andrew W. Huang, Elizabeth M. White, Richard A. Feifer, Carolyn Blackman, Christopher M. Santostefano, Yoojin Lee, Frank DeVone, Christopher W. Halladay, James L. Rudolph, Andrew R. Zullo, Vincent Mor, Stefan Gravenstein

**Affiliations:** Center of Innovation in Long-Term Services and Supports, Veterans Administration Medical Center, Providence, Rhode Island; Department of Health Services, Policy and Practice, School of Public Health, Brown University, Providence, Rhode Island; Department of Health Services, Policy and Practice, School of Public Health, Brown University, Providence, Rhode Island; Department of Health Services, Policy and Practice, School of Public Health, Brown University, Providence, Rhode Island; Department of Health Services, Policy and Practice, School of Public Health, Brown University, Providence, Rhode Island; Genesis HealthCare, Kennett Square, Pennsylvania; Genesis HealthCare, Kennett Square, Pennsylvania; Department of Health Services, Policy and Practice, School of Public Health, Brown University, Providence, Rhode Island; Department of Health Services, Policy and Practice, School of Public Health, Brown University, Providence, Rhode Island; Center of Innovation in Long-Term Services and Supports, Veterans Administration Medical Center, Providence, Rhode Island; Center of Innovation in Long-Term Services and Supports, Veterans Administration Medical Center, Providence, Rhode Island; Center of Innovation in Long-Term Services and Supports, Veterans Administration Medical Center, Providence, Rhode Island; Department of Health Services, Policy and Practice, School of Public Health, Brown University, Providence, Rhode Island; Center of Innovation in Long-Term Services and Supports, Veterans Administration Medical Center, Providence, Rhode Island; Department of Health Services, Policy and Practice, School of Public Health, Brown University, Providence, Rhode Island; Center of Innovation in Long-Term Services and Supports, Veterans Administration Medical Center, Providence, Rhode Island; Department of Health Services, Policy and Practice, School of Public Health, Brown University, Providence, Rhode Island; Center of Innovation in Long-Term Services and Supports, Veterans Administration Medical Center, Providence, Rhode Island; Department of Health Services, Policy and Practice, School of Public Health, Brown University, Providence, Rhode Island; Division of Geriatrics, Warren Alpert Medical School of Brown University, Providence, Rhode Island

## Abstract

**IMPORTANCE:**

A SARS-CoV-2 vaccine booster dose has been recommended for all nursing home residents. However, data on the effectiveness of an mRNA vaccine booster in preventing infection, hospitalization, and death in this vulnerable population are lacking.

**OBJECTIVE:**

To evaluate the association between receipt of a SARS-CoV-2 mRNA vaccine booster and prevention of infection, hospitalization, or death among nursing home residents.

**DESIGN, SETTING, AND PARTICIPANTS:**

This cohort study emulated sequentially nested target trials for vaccination using data from 2 large multistate US nursing home systems: Genesis HealthCare, a community nursing home operator (system 1) and Veterans Health Administration community living centers (VHA CLCs; system 2). The cohort included long-term (≥100 days) nursing home residents (10 949 residents from 202 community nursing homes and 4321 residents from 128 VHA CLCs) who completed a 2-dose series of an mRNA vaccine (either BNT162b2 [Pfizer-BioNTech] or mRNA-1273 [Moderna]) and were eligible for a booster dose between September 22 and November 30, 2021. Residents were followed up until March 8, 2022.

**EXPOSURES:**

Receipt of a third mRNA vaccine dose, defined as a booster dose (boosted group), or nonreceipt of a booster dose (unboosted group) on an eligible target trial date. If participants in the unboosted group received a booster dose on a later target trial date, they were included in the booster group for that target trial; thus, participants could be included in both the boosted and unboosted groups.

**MAIN OUTCOMES AND MEASURES:**

Test-confirmed SARS-CoV-2 infection, hospitalization, or death was followed up to 12 weeks after booster vaccination. The primary measure of estimated vaccine effectiveness was the ratio of cumulative incidences in the boosted group vs the unboosted group at week 12, adjusted with inverse probability weights for treatment and censoring.

**RESULTS:**

System 1 included 202 community nursing homes; among 8332 boosted residents (5325 [63.9%] female; 6685 [80.2%] White) vs 10 886 unboosted residents (6865 [63.1%] female; 8651 [79.5%] White), the median age was 78 (IQR, 68–87) years vs 78 (IQR, 68–86) years. System 2 included 128 VHA CLCs; among 3289 boosted residents (3157 [96.0%] male; 1950 [59.3%] White) vs 4317 unboosted residents (4151 [96.2%] male; 2434 [56.4%] White), the median age was 74 (IQR, 70–80) vs 74 (IQR, 69–80) years. Booster vaccination was associated with reductions in SARS-CoV-2 infections of 37.7% (95% CI, 25.4%–44.2%) in system 1 and 57.7% (95% CI, 43.5%–67.8%) in system 2. For hospitalization, reductions of 74.4% (95% CI, 44.6%–86.2%) in system 1 and 64.1% (95% CI, 41.3%–76.0%) in system 2 were observed. Estimated vaccine effectiveness for death associated with SARS-CoV-2 was 87.9% (95% CI, 75.9%–93.9%) in system 1; however, although a reduction in death was observed in system 2 (46.6%; 95% CI, −34.6% to 94.8%), this reduction was not statistically significant. A total of 45 SARS-CoV-2–associated deaths occurred in system 1 and 18 deaths occurred in system 2. For the combined end point of SARS-CoV-2–associated hospitalization or death, boosted residents in system 1 had an 80.3% (95% CI, 65.7%–88.5%) reduction, and boosted residents in system 2 had a 63.8% (95% CI, 41.4%–76.1%) reduction.

**CONCLUSIONS AND RELEVANCE:**

In this study, during a period in which both the Delta and Omicron variants were circulating, SARS-CoV-2 booster vaccination was associated with significant reductions in SARS-CoV-2 infections, hospitalizations, and the combined end point of hospitalization or death among residents of 2 US nursing home systems. These findings suggest that administration of vaccine boosters to nursing home residents may have an important role in preventing COVID-19–associated morbidity and mortality.

## Introduction

Nursing home residents have experienced substantial SARS-CoV-2–associated morbidity and mortality.^1,2^ Resurgence with the Delta variant in the late summer and early autumn of 2021 and waning immunity among nursing home residents^3^ led the Centers for Disease Control and Prevention (CDC) to recommend a booster dose for those vaccinated with a 2-dose mRNA series.^4,5^ The US Food and Drug Administration (FDA) amended the emergency use authorizations for the BNT162b2 vaccine (Pfizer-BioNTech) in September 2021 and the mRNA-1273 vaccine (Moderna) in October 2021. The FDA recommended a booster dose at least 6 months after receipt of the second dose of an mRNA vaccine for individuals at high risk, including those 65 years or older.^6^ Both mRNA vaccines were also authorized in August 2021 to be used for additional doses in immunocompromised individuals, which applied to many older adults in long-term care facilities. As of February 1, 2022, approximately 66% of US nursing home residents had received a booster dose.^6^

Despite the high risk of SARS-CoV-2 morbidity and death among nursing home residents, limited data exist on the effectiveness of boosters in mitigating infection or death in this vulnerable population. Vaccine trials did not include nursing home residents, and timely patient-level observational data are not widely available. Nursing homes are an optimal environment for measurement of vaccine effectiveness (VE) because of residential stability, systematic documentation of immunizations (including boosters), and frequent testing for SARS-CoV-2. Additional clinical evidence of booster effectiveness may support efforts to increase booster distribution for this vulnerable population.

To address this gap, we used electronic health record (EHR) data updated daily from 2 large multistate long-term care systems. Using a nested target trial emulation method,^7^ we estimated the VE of an mRNA booster dose (vs no booster dose) among residents who completed the primary series of an mRNA vaccine. Our outcomes included any laboratory-confirmed SARS-CoV-2 infection, hospitalization, or death. We hypothesized that receipt of an mRNA vaccine booster would be associated with lower risk of these outcomes.

## Methods

### Study Design and Population

This cohort study was designed to emulate a sequence of nested target trials comparing the estimated additional effectiveness of a SARS-CoV-2 mRNA vaccine booster vs the primary vaccine series only among nursing home residents. We planned a priori to analyze 2 cohorts identified in separate health systems and to report results separately. The primary reason for separate cohorts was that the data use agreements did not allow exporting and pooling of person-level data outside of the secure server environment. The first system included 202 nursing homes in 19 states, with a heavy concentration in the northeastern region, operated by Genesis HealthCare (system 1). The second included veterans residing in 128 community living centers (CLCs; analogous to nursing homes) spread nationwide and managed by the Veterans Health Administration (VHA; system 2). The study emulated daily (Monday through Friday) target trials from September 22 through November 30, 2021. September 22 was the date on which the FDA authorized the BNT162b2 vaccine booster for use in nursing home residents. November 30, 2021, was selected to allow at least 12 weeks of follow-up observation time to March 8, 2022. Data were collected from administrative and clinical records in both systems. This study was deemed exempt from informed consent by the institutional review board of Brown University, and the VHA project was approved by the institutional review board of the Providence VA Medical Center. The study followed the Strengthening the Reporting of Observational Studies in Epidemiology (STROBE) reporting guideline for cohort studies.

The target trial method involves defining a target trial design and emulating the trial in the observational data.^7,8^ Specific dates are selected as trial dates on which those who meet eligibility criteria and are assigned or not assigned to receive treatment are included. Details about our target trial for both nursing home systems are available in Table 1; this table shows how the trial was emulated in the observational data. On each day, residents were considered eligible for inclusion if they (1) had been present in the nursing home for at least 100 days, with a gap of no more than 10 days (ie, residents could leave and come back, but not for >10 days), and (2) had received a 2-dose mRNA vaccine series at least 120 days earlier. Residents who received the 1-dose Ad26.COV2.S vaccine (Janssen/Johnson & Johnson) were excluded due to small numbers. Residents were also excluded if they had a SARS-CoV-2 infection in the previous 90 days, planned discharge according to their most recent assessment, received treatment with monoclonal antibodies in the previous 90 days, were receiving hospice care, or had already received a third vaccine dose before the index date. After exclusions, the total sample included long-term (≥100 days) nursing home residents (10 949 residents from 202 community nursing homes and 4321 residents from 128 VHA CLCs). Residents were defined as boosted if they received a booster vaccine dose on the index date (boosted group); residents who met all eligibility criteria but did not receive a booster vaccine dose on that day were defined as unboosted (unboosted group).

### Study Outcomes and Covariates

We examined 4 postbooster outcomes defined a priori: (1) any incident SARS-CoV-2 infection, (2) hospitalization, (3) death, and (4) combined end point of hospitalization or death. All study outcomes were obtained via the EHR from the 2 systems.

SARS-CoV-2 infection was defined by a positive result on a SARS-CoV-2 rapid antigen or polymerase chain reaction test occurring after the index trial date and with no test confirming infection in the previous 90 days. Testing results were extracted from laboratory results in both systems.^9^

SARS-CoV-2–associated hospitalization was defined as transfer to an acute care hospital occurring within 21 days of a positive SARS-CoV-2 test. Transfers were identified through census records in system 1 and bed section codes identifying acute care episodes in system 2, which had a single EHR system for both nursing homes and hospitals.

SARS-CoV-2–associated death was defined as death occurring within 30 days of a positive SARS-CoV-2 test. Deaths were identified through transfers to funeral homes or death records in the daily census in system 1 and through a combination of census and vital status records in system 2.

Immunization records in each EHR were used to identify primary vaccination and booster events.^10^ We indexed additional resident covariates separately for each eligible trial date (ie, a person’s baseline covariates may have differed for each trial because each trial represented a different index date). Data were obtained from the EHR in both systems. In system 1, additional information from the nursing home Minimum Data Set (MDS) assessments were included.^11^ The MDS includes demographic characteristics (age, sex, race and ethnicity, and language), clinical diagnoses, MDS measures of cognitive and physical function, an MDS indicator of limited life expectancy, previous SARS-CoV-2 infections, and influenza vaccination for the current season up to the index date. The data in system 2 did not include MDS assessments and relied on demographic characteristics and *International Classification of Diseases, Tenth Revision, Clinical Modification* diagnosis codes in the EHR (recorded in the 12 months before the index date) for chronic condition information. These data were classified using the Elixhauser comorbidity classification system.^12^ In both systems, information on resident race and ethnicity was documented by clinicians and included in our analysis due to the potential of race and ethnicity to be confounders of vaccination and outcomes. We characterized residents as immunocompromised if they had received an immunosuppressive medication or had a diagnosis for a qualifying condition (details are available in eTable 1 in Supplement 1). We also measured overall SARS-CoV-2 testing rates at the facility, SARS-CoV-2 positivity rates in the period before each index date (as a measure of local disease risk), and facility-level factors, such as a quality of care and infection control.

### Statistical Analysis

A brief discussion of the target trial emulation method is available in the eAppendix in Supplement 1. The target trial emulation approach has been increasingly used to address challenges of immortal time and selection bias in observational data. It is well suited to studying VE in situations in which the unvaccinated group lacks an obvious starting date for follow-up. For each index date, residents meeting eligibility criteria (Table 1) were included. If residents received a booster vaccine dose on a target trial date, they were assigned to the booster arm. After residents received a booster dose, they were ineligible for future trial dates; therefore, data from the boosted arm did not contain repeated observations. Eligible residents who did not receive a booster dose were included in the control arm. If residents were eligible on multiple dates (eg, if they never received a booster), we randomly selected 1 eligible target trial date for analysis.^7^ The random selection of dates for the control group only determined which target trial date was used and the total amount of follow-up time from that starting date; all individuals in the control group were included.

From each trial date, survival time was computed as the minimum of either time to outcome or time to a censoring event (ie, death or last available date of follow-up [March 8, 2022]). In community nursing homes, residents were censored at transfer because only the nursing home EHR was available. However, VHA facilities captured clinical care outside of CLCs, so transfer outside the CLC was not a censoring event. Residents in the control group were censored if they received a booster dose after the index date but contributed follow-up time until the date of booster administration. If residents in the control group received a booster dose on a later target trial date, they were included in the boosted group for that target trial (thus, residents could be included in both arms).

The same analytical approach was used for both cohorts, but analysis was completed separately by separate analysts (F.D. and C.W.H.). Each event was modeled using pooled logistic regression analysis, with cumulative incidence (ie, risk) measured as 1 minus the probability of event-free survival at each time point (weeks from time 0). The relative ratio of the cumulative incidence curves between groups (boosted vs unboosted) at each time point was computed, with 1 minus relative risk reported as the conventional VE statistic. The cumulative incidence at week 12 was selected as the primary follow-up time of interest for reporting. Final statistical models were adjusted for confounding and informative censoring with inverse probability weighting. Final model covariates and coefficients for each health care system are shown in eTable 4 and eTable 5 in Supplement 1.

Model selection for weights was based on a priori experience with confounders (ie, local COVID-19 rates) and observed baseline imbalances in covariates, which were plausibly common factors associated with treatment and outcome. Absolute standardized mean differences of less than 0.1, categorized by booster status, were used to guide acceptable balance between groups after adjustment. Adjusted models were evaluated for observations with nonmissing data and no imputation. The final weight was a product of treatment and censoring weights. Weights were truncated at their 99% upper quantile. Sampling with replacement by resident (ie, bootstrapping) with 1000 replications was used to generate 95% CIs accounting for the estimated probability weights and crossover of treatment groups by resident. Initial data preparation was conducted using SAS software, version 9.4 (SAS Institute Inc), and Stata software, version 16 (StataCorp LLC). All analyses were performed using R statistical software, version 4.0.1 (R Foundation for Statistical Computing).

## Results

### Study Population

System 1 included 202 community nursing homes; among 8332 boosted residents (5325 [63.9%] female; 6685 [80.2%] White) vs 10 886 unboosted residents (6865 [63.1%] female; 8651 [79.5%] White), the median age was 78 (IQR, 68–87) years vs 78 (IQR, 68–86) years. System 2 included 128 VHA CLCs; among 3289 boosted residents (3157 [96.0%] male; 1950 [59.3%] White) vs 4317 unboosted residents (4151 [96.2%] male; 2434 [56.4%] White), the median age was 74 (IQR, 70–80) vs 74 (IQR, 69–80) years. Resident counts by inclusion and exclusion criteria for each day are shown in eTable 2 and eTable 3 in Supplement 1.

A resident flowchart and information about assignment to each group are provided in eFigure 1 in Supplement 1. More residents in system 1 received a BNT162b2 (Pfizer-BioNTech) booster dose (5616 of 8332 individuals [67.4%]) than residents in system 2 (1433 of 3289 individuals [43.6%]). The follow-up time contributed was 1 163 155 days (median [IQR], 37 [13–117] days per resident) in system 1 and 356 049 days (median [IQR], 41 [11–84] days per resident) in system 2.

Other baseline characteristics of both cohorts, including the boosted and unboosted groups, are shown in Table 2. Boosted vs unboosted residents differed greatest by length of stay (system 1: median [IQR], 764 [428–1279] days vs 698 [355–1210] days; system 2: median [IQR], 761 [378–1558] days vs 694 [273–1413] days) and time since second vaccination (system 1: median [IQR], 265 [252–277] days vs 248 [232–260] days; system 2: median [IQR], 270 [252–285] days vs 260 [252–280] days).The balance across covariates after inverse probability weights were applied is shown in eFigure 2 in Supplement 1. Compared with boosted residents in system 2, those in system 1 were older (median [IQR], 78 [68–87] years vs 74 [70–80] years), less likely to be of Black race (857 of 8332 individuals [10.3%] vs 731 of 3289 individuals [22.3%]) and more likely to be female (5325 of 8332 individuals [63.9%] vs 132 of 3289 individuals [4.0%]).

### Estimated Vaccine Effectiveness of Booster Dose

**Figure 1.**
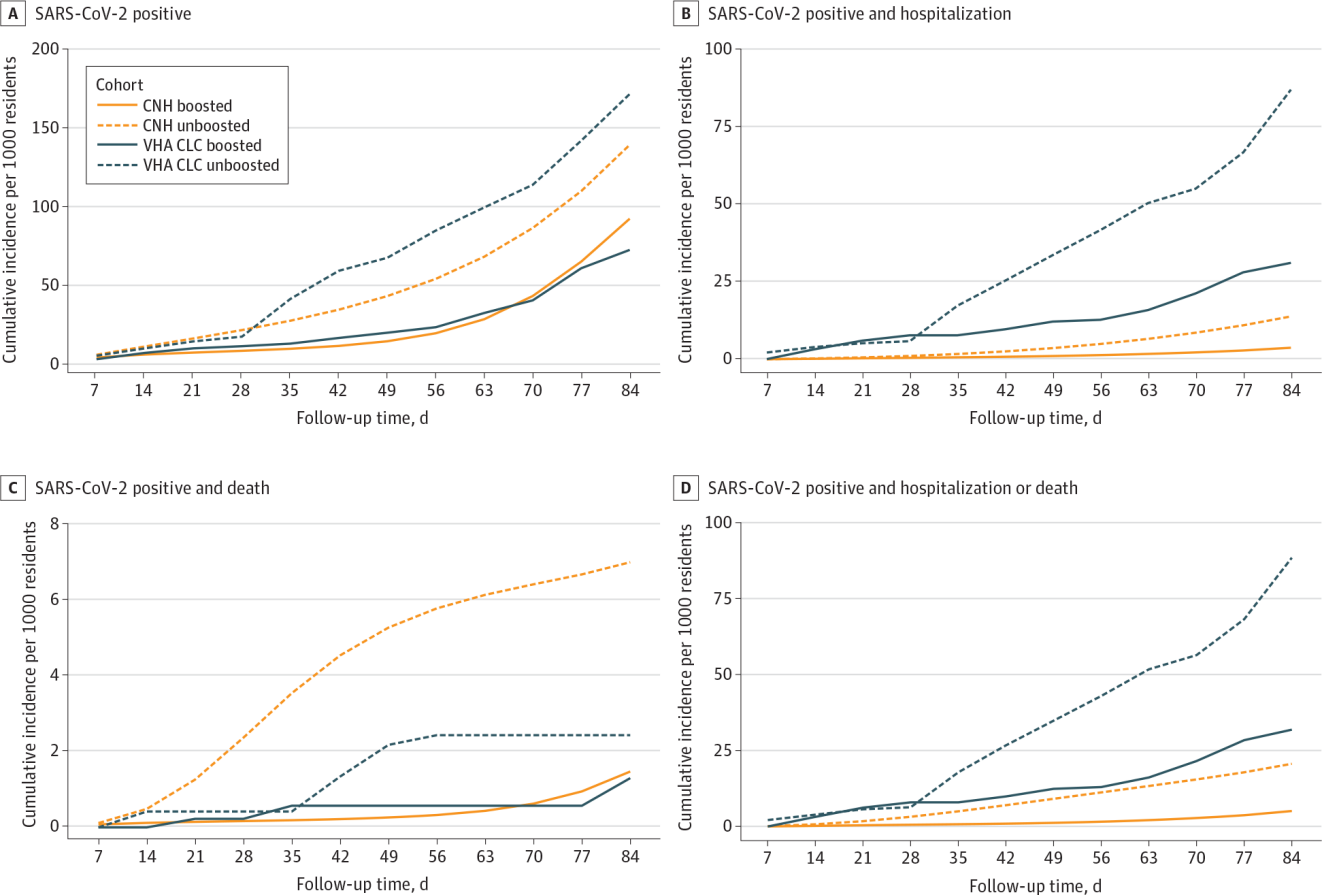
SARS-CoV-2 Cumulative Incidence in 2 US Nursing Home Systems by Booster Status Among nursing home residents eligible to receive an mRNA vaccine booster dose between September 22 to November 30, 2021. Days of follow-up were measured from the index date of the target trial. A total of 202 community nursing homes (CNHs) and 128 Veterans Health Administration community living centers (VHA CLCs) were included.

Cumulative incidence curves for the 4 COVID-19 outcomes up to 12 weeks are shown in Figure 1. Overall, compared with boosted residents in system 2, those in system 1 had higher rates of SARS-CoV-2 infection over 12 weeks (100.1 [95% CI, 92.8–107.3] infections/1000 residents vs 72.5 [95% CI, 62.4–81.9] infections/1000 residents) and SARS-CoV-2–associated death (1.4 [95% CI, 0.8–2.2] deaths/1000 residents vs 1.3 [95% CI, 0.2–2.6] deaths/1000 residents) but lower rates of SARS-CoV-2–associated hospitalization (3.9 [95% CI, 2.7–5.2] hospitalizations/1000 residents vs 31.2 [95% CI, 25.0–38.0] hospitalizations/1000 residents) associated with SARS-CoV-2. A total of 45 SARS-CoV-2–associated deaths were observed in system 1 vs 18 SARS-CoV-2–associated deaths in system 2.

At week 12, booster vaccination was associated with reductions in the risk of SARS-CoV-2 infection of 37.7% (95% CI, 25.4%–44.2%) in system 1 and 57.7% (95% CI, 43.5%–67.8%) in system 2 (Table 3). The estimated VE for SARS-CoV-2–associated hospitalization was 74.4% (95% CI, 44.6%–86.2%) in system 1 and 64.1% (95% CI, 41.3%–76.0%) in system 2. In system 1, the VE for SARS-CoV-2–associated death was 87.9% (95% CI, 75.9%–93.9%); in system 2, a reduction in death was observed (46.6%; 95% CI, −34.6% to 94.8%), but this reduction was compatible with chance. The VE for the combined end point of hospitalization or death was 80.3% (95% CI, 65.7%–88.5%) in system 1 vs 63.8% (95% CI, 41.4%–76.1%) in system 2.

**Figure 2.**
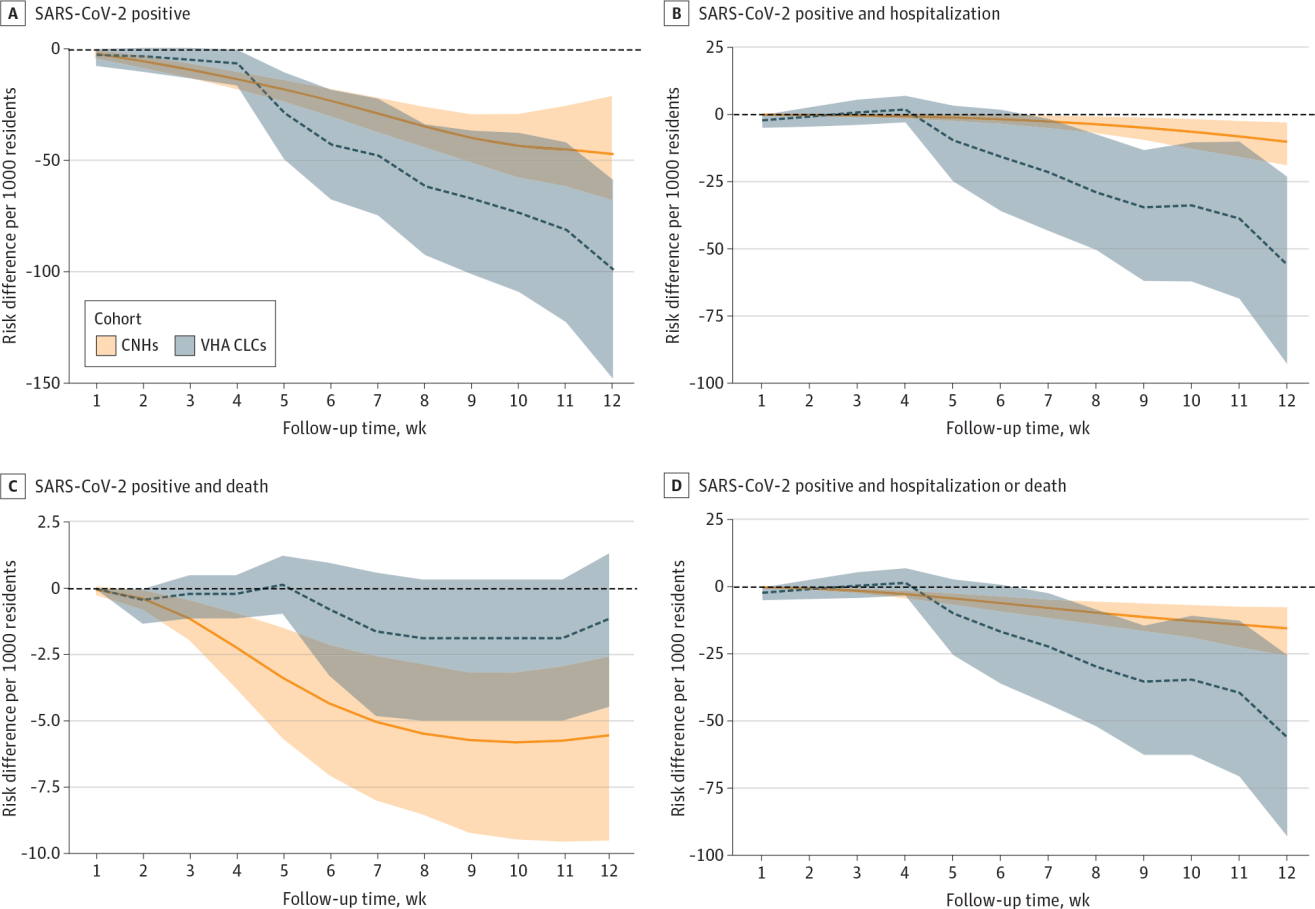
Estimated Vaccine Effectiveness for Absolute Risk of Outcomes Associated With SARS-CoV-2 Among Residents Receiving Booster Doses in 2 US Nursing Home Systems Among residents eligible to receive an mRNA vaccine booster dose between September 22 to November 30, 2021. A total of 202 community nursing homes (CNHs) and 128 Veterans Health Administration community living centers (VHA CLCs) were included. Shading represents 95% CIs.

The estimated VE in absolute differences is shown in Figure 2. The estimated VE in each system differed mostly in hospitalization rates. We estimated that 47 residents in system 1 and 18 residents in system 2 needed to receive a booster dose to prevent 1 SARS-CoV-2–associated hospitalization or death. Relative VE is provided in eFigure 2 in Supplement 1.

## Discussion

This cohort study estimated the clinical effectiveness of an mRNA SARS-CoV-2 vaccine booster among 2 large multistate populations of nursing home residents. We sought to estimate VE among those eligible for our hypothetical target trial. We found that residents in system 1 who received a booster dose had a substantially lower risk of SARS-CoV-2 infection, hospitalization, and death. Residents in system 2 had a similarly lower risk of infection and hospitalization; however, risk of death was similar in boosted vs unboosted residents in this system, possibly because only 18 COVID-19–associated deaths occurred in the observed time period. With the exception of the outcome of SARS-CoV-2–associated deaths in system 2, all measured outcomes were associated with significantly lower risk in boosted vs unboosted residents. Although the estimated VE varied somewhat by nursing home system (community nursing homes vs VHA CLCs), the findings between cohorts were consistent overall; residents who received a booster dose had a significantly lower risk of SARS-CoV-2 infection and morbidity (as measured by SARS-CoV-2–associated hospitalization).

To evaluate the robustness of VE, we examined 2 substantially different nursing home populations: a large private US operator providing nursing home care and a national health care system caring specifically for older and/or disabled veterans. These 2 health systems were demographically different (eg, few women were present in VHA CLCs). Notably, the rate of hospitalization was substantially higher in VHA CLCs vs community nursing homes (31.2 hospitalizations/1000 residents vs 3.9 hospitalizations/1000 residents over 12 weeks). The rates and reasons for acute hospitalization may differ for non–COVID-19 related differences in the resident comorbidity burden and differences in the availability of hospice care, advance planning, and clinician practice regarding hospitalization. It should be noted that the sample size of system 2 (VHA CLCs) was 39.5% the size of the sample in system 1 (community nursing homes), with shorter follow-up time and fewer facilities. However, consistent VE findings in these 2 disparate cohorts suggest the importance of boosters for the overall US nursing home population.

There are few other studies of booster effectiveness in nursing home residents to date. A recent study published in the *Morbidity and Mortality Weekly Report*^13^ evaluated publicly reported data for more than 15 000 US nursing homes and estimated a 46% VE for preventing SARS-CoV-2 infection, which was similar to our VE estimate; however, the researchers did not report results for death or hospitalization. Bar-On et al^14^ reported observational data from Israeli community-dwelling adults 60 years and older who received a booster dose 5 months after completing the primary vaccine series. Comparing confirmed infections and adjusting for person-days at risk, older adults had approximately 88% fewer confirmed infections in the 12 to 42 days after receipt of the booster dose,^14^ during a period in which the Delta variant was the predominant virus in circulation. During the same period, Muhsen at al^15^ reported on more than 41 000 Israeli nursing home residents who received a booster dose approximately 5 months after completing the primary series compared with community-dwelling adults. The boosted nursing home residents had a 71% lower rate of infection and an 80% lower rate of hospitalization than unboosted community dwellers.^15^ A substantial limitation of this previous work is that it lacked extrapolation to the Omicron variant (BA.4 and BA.5 strains), which was not yet circulating; however, our analysis included the early period of Omicron circulation through February 2022. We found a sustained protective benefit up to at least 3 months in persons receiving a booster dose vs persons not receiving a booster dose. This finding was consistent with other work^16,17^ suggesting a third dose of an mRNA vaccine offered protection against Omicron variants vs 2 doses alone.

The present work evaluated VE by using an emulated trial design, which allowed us to include unboosted participants with characteristics comparable with those of boosted participants who differed primarily by the date on which they received a SARS-CoV-2 vaccine rather than by indication, baseline covariates, or exposure risk to circulating strains. The study included residents in facilities across broad geographic and socioeconomic distributions. Differences in our estimates vs others could arise from differences in methods, population health, virus characteristics, or other reasons.

### Limitations

This study has several limitations. Vaccination status was not randomly assigned, so our observational results are potentially limited by unmeasured confounding. The relatively small differences between groups are encouraging and may be explained by the eligibility requirement of primary series vaccination, resulting in greater cohort homogeneity. In addition, the cumulative incidence rates by group in the first week reveal similar disease risk. This similarity suggests a relatively low risk of residual confounding if the 2 groups had similar baseline event risk in the period after vaccination but before developing postbooster immunity. We did not account for whether the overall facility vaccination rate impacts individual VE (ie, interference). A 12-week follow-up period was selected because recommendations for a fourth dose began in March 2022, which complicated follow-up of residents (ie, some would receive another vaccine). In addition, deaths in the community nursing homes (system 1) are clearly recorded; however, for residents who die after transfer, records are more sporadic. System 2 (VHA CLCs) links deaths to vital statistics files and does not have this limitation. This discrepancy is the reason a composite end point, in which the outcome is hospitalization or death, is reported. We could not evaluate effectiveness against specific variants, including currently circulating strains like BA.4 and BA.5, because genotyping was not routinely collected in this clinical practice setting.

## Conclusions

This cohort study found lower risk of SARS-CoV-2 infection and hospitalization after booster adoption by 2 large US nursing home systems during a period in which Delta and Omicron variants were circulating. These findings suggest the importance of SARS-CoV-2 booster vaccination for the nursing home population.

## Supplementary Material

Supplement 2 Data Sharing Statement

Supplement 1 **eAppendix.** Details on Analytical Approach and Statistical Analysis
**eFigure 1.** Flowchart of Eligibility for Target Trials of mRNA Boosters in 2 US Nursing Home Systems
**eFigure 2.** Covariate Balance Preprobability and Postprobability Weights
**eTable 1.** Definition of Immunocompromised Status
**eTable 2.** System 1 Trial Eligibility by Date
**eTable 3.** System 2 Trial Eligibility by Date
**eFigure 3.** Relative Booster Effectiveness in First 12 Weeks Among Nursing Home Residents
**eTable 4.** Coefficients From Model Estimating Inverse Probability of Treatment Weights
**eTable 5.** Coefficients From Model Estimating Inverse Probability of Censoring Weights

## Figures and Tables

**Table 1.** Protocol of the Target Trial to Study the Estimated Effectiveness of Booster Vaccination in Community Nursing Homes and Veterans Health Administration Community Living Centers^a^

Component	Target trial protocol	Consistency with target trial protocol	
		System 1: community nursing homes	System 2: VHA community living centers
Eligibility	Inclusion criteria: Present in nursing home on any index date between September 22 and November 20, 2021 Admitted ≥100 d before index date Received fullmRNA primary vaccine series (2 doses at >14 days between doses) ≥120 d before index date Exclusion criteria: Had a medicalhistory of SARS-CoV-2 infection <90 d before index date (any *ICD-10-CM* diagnosis code or positive result on PCR test) Received treatment with monoclonal antibodies ≤90 d before index date Already received SARS-CoV-2 booster dose before index date Receiving hospice care	Additions to protocol: Complete-case analysis (excluding missing data) Nursing home operated by Genesis HealthCare as of December 18, 2021 No plan for discharge	Addition to protocol: Long stay of 90 d before vs 100 d before with gap of 10 d
Treatment	Booster dose (third dose >6 mo after completion of primary series) vs placebo	Deviation from protocol: Comparison with booster dose received by control group rather than placebo	Same as protocol
Assignment	Random assignment byindividual	Deviations from protocol: Nonrandomized assignment Use of inverse probability weighting to adjust for bias	Same as protocol
Follow-up	Censoring events: Death associated with any cause Transfer out of nursing home to hospital or other facility	Addition to protocol: Receipt of booster dose in control group	Deviation from protocol: Transfer out of VA institutional care to community care
Outcome	Positive PCR or antigen test result for SARS-CoV-2 infection and SARS-CoV-2-associated events (infection, hospitalization, or death)	Same as protocol	Same as protocol
Statisticalanalysis	2 analyses: Cumulative incidence at specific time points Vaccine effectiveness at wk 12	Same as protocol	Same as protocol

Abbreviations: *ICD-10-CM, International Classification of Diseases, Tenth Revision, Clinical Modification;* PCR, polymerase chain reaction; VHA, Veterans Health Administration.

a The aim was to evaluate the relative risk of SARS-CoV-2 infection among those who received a booster dose vs a primary series of a vaccine only. Column 2 shows the hypothetical design of a randomized clinical trial involving nursing home residents. Columns 3 and 4 show concordance, deviation, and additions to the target trial protocol for each system.

**Table 2.** Baseline Characteristics of Nursing Home Residents by Booster Status and Nursing Home System^a^

Characteristic	Participants, No. (%)			
	System 1: community nursing homes^b^		System 2: VHA community living centers^c^	
	Unboosted group (n = 10886)	Boosted group (n = 8332)	Unboosted group (n = 4317)	Boosted group (n = 3289)
Age, median (IQR), y	78 (68–86)	78 (68–87)	74(69–80)	74(70–80)
Sex				
Male	4021 (36.9)	3007 (36.1)	4151 (96.2)	3157 (96.0)
Female	6865 (63.1)	5325 (63.9)	166(3.8)	132 (4.0)
Preindex length of stay, median (IQR), d	698 (355–1210)	764(428–1279)	694(273–1413)	761 (378–1558)
Race and ethnicity				
Black	1141 (10.5)	857 (10.3)	962 (22.3)	731 (22.2)
Hispanic	574(5.3)	423 (5.1)	184 (4.3)	134(4.1)
White	8651 (79.5)	6685 (80.2)	2434(56.4)	1950 (59.3)
Other^d^	522 (4.8)	369(4.4)	737 (17.1)	474(14.4)
Comorbidities				
Current smoker	371 (3.4)	275 (3.3)	548 (12.7)	368 (11.2)
Alzheimer disease and related dementias	5319 (48.9)	4242 (50.9)	2871 (66.5)	2217 (67.4)
Immunocompromised	1258 (11.6)	1006 (12.1)	1254(29.0)	916(27.9)
Pulmonary disease	2514(23.1)	1880 (22.6)	1380 (32.0)	1043 (31.7)
Diabetes (any type)	4205 (38.6)	3205 (38.5)	2124(49.2)	1613 (49.0)
Paralysis	1614(14.8)	1241 (14.9)	1058 (24.5)	756 (23.0)
Time from second dose to booster dose, median (IQR), d	248 (232–260)	265(252–277)	260 (252–280)	270 (252–285)
No. of SARS-CoV-2 tests, mean (SD)^e^				
Past 14 d	0.5 (1.7)	0.5 (1.7)	1.9 (1.7)	19 (1.7)
Past 90 d	2.9 (7.7)	3.4(8.7)	11.8(8.6)	11.9(8.7)

Abbreviation: VHA, Veterans Health Administration.

a Among nursing home residents eligible to receive an mRNA vaccine booster dose between September 22 and November 30, 2021. All diagnoses were as defined by the Charlson Comorbidity Index and identified via codes from the *International Classification of Diseases, Tenth Revision, Clinical Modification*.

b Includes 202 community nursing homes.

c Includes 128 VHA community living centers.

d Includes American Indian, Asian, Pacific Islander, and unreported race and/or ethnicity.

e Includes all tests received, regardless of positive or negative results.

**Table 3.** Estimated Vaccine Effectiveness at 12Weeks Among Nursing Home Residents Who Did vs Did Not Receive a Booster Dose^a^

Outcome	Cumulative incidence per 1000 residents (95% CI)		Relative vaccine effectiveness, % (95% CI)^b^	Risk difference in cumulative incidence (95% CI)
	Boosted group	Unboosted group		
**Infection**				
System 1: CNHs	100.1 (92.8 to 107.3)	160.5 (133.2 to 179.9)	37.7 (25.4 to 44.2)	−60.4 (−72.5 to −40.4)
System 2: VHACLCs	72.5 (62.4 to 81.9)	171.2 (127.9 to 216.2)	57.7 (43.5 to 67.8)	−98.8 (−146.0 to−57.0)
**Hospitalization**				
System 1: CNHs	3.9 (2.7 to 5.2)	15.1 (7.7 to 24.4)	74.4 (44.6 to 86.2)	−11.2 (−19.3 to −4.9)
System 2: VHA CLCs	31.2 (25.0 to 38.0)	86.9 (54.8to 121.8)	64.1 (41.3 to 76.0)	−55.8 (−93.0 to−23.0)
**Death**				
System 1: CNHs	1.4 (0.8 to 2.2)	11.3 (6.5 to 16.9)	87.9 (75.9to 93.9)	−9.9 (−14.7 to−5.7)
System 2: VHA CLCs	1.3 (0.2 to 2.6)	2.4 (0.3 to 5.5)	46.6 (−34.6 to 94.8)	−1.1 (−4.5 to 1.3)
**Composite outcome (hospitalization or death)**				
System 1: CNHs	5.2 (3.8 to 6.6)	26.3 (15.9 to 38.3)	80.3 (65.7 to 88.5)	−21.1 (−31.6 to −12.2)
System 2: VHA CLCs	31.8 (25.4 to 38.9)	87.8 (56.2 to 123.8)	63.8 (41.4 to 76.1)	−56.1 (−93.2 to−24.8)

Abbreviations: VHA CLC, Veterans Health Administration community living center; CNH, community nursing home.

a Among nursing home residents eligible to receive an mRNA vaccine booster dose between September 22 and November 30, 2021. Includes residents from 202 CNHs and 128 VHA CLCs.

b Relative vaccine effectiveness was calculated as 1 minus the cumulative incidence ratio multiplied by 100.

## Data Availability

See Supplement 2.

## Key Points

### Question

What is the estimated vaccine effectiveness up to 12 weeks for the first COVID-19 mRNA booster vaccines administered in US nursing homes?

### Findings

In this cohort study of 10 949 residents of 202 community nursing homes and 4321 residents of 128 Veterans Health Administration community living centers, booster vaccination was associated with significant reductions in SARS-CoV-2 infections, hospitalizations, and the combined end point of hospitalizations or deaths.

### Meaning

These findings suggest that administration of a SARS-CoV-2 mRNA vaccine booster among nursing home residents may have played an important role in preventing COVID-19–associated morbidity and mortality.
