# Clarification of Calculation Methods Added to Table

**DOI:** 10.1001/jamanetworkopen.2023.52991

**Published:** 2024-01-04

In the Original Investigation titled “Infections, Hospitalizations, and Deaths Among US Nursing Home Residents With vs Without a SARS-CoV-2 Vaccine Booster,”^1^ published December 7, 2022, a footnote has been added to Table 3 clarifying how cumulative incidence was calculated. This article has been corrected.^1^
