# Supplementary material for: Infections, Hospitalizations, and Deaths Among US Nursing Home Residents With vs Without a SARS-CoV-2 Vaccine Booster
Source: JAMA Netw Open. Author manuscript; Available in PMC 2023 Jan 27. (PMC9856563; doi:10.1001/jamanetworkopen.2022.45417)
Supplement: Supplement 2 — Data Sharing Statement [file NIHMS1864106-supplement-Supplement_2.pdf]

Data Sharing Statement

McConeghy. Infections, Hospitalizations, and Deaths Among US Nursing Home Residents With vs Without a SARS-CoV-2 Vaccine Booster. *JAMA Netw Open*. Published December 07, 2022. doi:10.1001/jamanetworkopen.2022.45417

Data
Data available: No

Additional Information
Explanation for why data not available: Sharing data is not permitted under existed DUA and includes personal health information
