# Supplementary material for: Infections, Hospitalizations, and Deaths Among US Nursing Home Residents With vs Without a SARS-CoV-2 Vaccine Booster
Source: JAMA Netw Open. Author manuscript; Available in PMC 2023 Jan 27. (PMC9856563; doi:10.1001/jamanetworkopen.2022.45417)
Supplement: Supplement 1 — eAppendix. Details on Analytical Approach and Statistical Analysis eFigure 1. Flowchart of Eligibility for Target Trials of mRNA Boosters in 2 US Nursing Home Systems eFigure 2. Covariate Balance Preprobability and Postprobability Weights eTable 1. Definition of Immunocompromised Status eTable 2. System 1 Trial Eligibility by Date eTable 3. System 2 Trial Eligibility by Date eFigure 3. Relative Booster Effectiveness in First 12 Weeks Among Nursing Home Residents eTable 4. Coefficients From Model Estimating Inverse Probability of Treatment Weights eTable 5. Coefficients From Model Estimating Inverse Probability of Censoring Weights [file NIHMS1864106-supplement-Supplement_1.pdf]

Supplemental Online Content

McConeghy KW, Bardenheier B, Huang AW, et al. Infections, hospitalizations, and deaths among us nursing home residents with vs without a SARS-CoV-2 vaccine booster. *JAMA Netw Open*. 2022;5(12):e2245417. doi:10.1001/jamanetworkopen.2022.45417

eAppendix. Details on Analytical Approach and Statistical Analysis

eFigure 1. Flowchart of Eligibility for Target Trials of mRNA Boosters in 2 US Nursing Home Systems

eFigure 2. Covariate Balance Before and After Weighting

eTable 1. Definition of Immunocompromised Status

eTable 2. System 1 Trial Eligibility by Date

eTable 3. System 2 Trial Eligibility by Date

eFigure 3. Relative Booster Effectiveness in First 12 Weeks Among Nursing Home Residents

eTable 4. Coefficients From Model Estimating Inverse Probability of Treatment Weights

eTable 5. Coefficients From Model Estimating Inverse Probability of Censoring Weights

This supplemental material has been provided by the authors to give readers additional information about their work.

© 2022 McConeghy KW et al. *JAMA Network Open*.

eAppendix. Details on Analytical Approach and Statistical Analysis

Note

System 1: Residents from a large private provider of long-term care including 202 facilities concentrated in the northeast.

System 2: Veteran residents from 127 community-living centers administered by the Veterans Healthcare Administration

Further explanation of the target trial method

This study approach can be described as an emulation of sequential target trials. The study first defines the parameters of the target trial (Table 1), then emulates these parameters as closely as possible in the observational data. For the target trial, an index date is selected which represents the “time zero” which is referenced for all the historical covariates (i.e. time since second dose), and for assigning follow-up and identifying subsequent censoring or outcome events. If the study used a single index date, only a small number would be vaccinated on that given day. Typically for target trial emulations, we define a series of sequential index dates, from the starting period of giving a booster dose, September, 22, 2021 through the end of November 30, 2021. Weekends are excluded because few vaccinations are given on weekends.

When a resident receives a booster vaccination on a target trial date, they are “assigned” to the booster arm. Once a resident is boosted, they are ineligible for future trial dates because one criteria is not already being boosted (see Table 1 and consort diagram in Figure 1). Therefore, a person only appears once in the booster arm. Alternatively, residents might be eligible as controls on multiple emulated trial dates up until they received a booster. If these repeated observations by person are pooled together it is more statistically efficient, but also requires greater computational effort because of inflated sample size and the bootstrapping procedure. Because we had a reasonably large sample, and wished to avoid very long computation times (days to weeks), we randomly selected one eligible target trial date per control resident. This doesn’t impact the total number of unique persons in the study, or which events are counted, but randomly selects a “time zero” for each control person. Controls are censored if they subsequently receive a booster dose, but contribute follow-up time until the point of censoring on the date of booster administration. If that control person is otherwise eligible and the date they receive a booster is a target trial date, they will be included in the treatment arm as well. Persons are unique within assignment arm (boosted or unboosted), but can appear in both arms of the study. This is accounted for in the statistical analysis by bootstrapping, where the resampling occurs at the person-level cluster.

Because actual assignment of vaccine on a given day is non-random, inverse probability of treatment weights are used to weight the sample and create a pseudopopulation of observations where probability of assignment to vaccine is similar between groups. Additionally, because control residents censor for treatment, this (by design) informative censoring between groups must be accounted for. We use inverse probability weighting for the probability of remaining uncensored to account for this mechanism.

Why employ this method versus traditional retrospective analytical methods?

© 2022 McConeghy KW et al. *JAMA Network Open*.

The reason the authors elected for this method was for two reasons: 1) Target trial emulation is a helpful tool for refining your study question and identifying a causal contrast of interest, with clearly laid out inclusion/exclusion criteria for your cohort, 2) There is an inherent challenge of comparing persons receiving a vaccine versus non-receipt. If not carefully addressed immortal time bias can be introduced, where there exists a misalignment of eligibility assessment, start of follow-up and assignment of treatment periods between groups. Target trial emulation is useful because it helps pinpoint eligibility assessment and treatment assignment on at the same timepoint and avoids this bias. There are many problems in observational data, unmeasured confounding, model misspecification etc. that target trial emulation doesn't solve but it is a useful and practical tool for causal inference.

Statistical analysis

We estimated two weights for our statistical analysis, the first were stabilized inverse probability of treatment weights (IPTW). This was the probability of receiving the vaccine on each target trial date, adjusting for measured baseline confounders. A stabilized propensity score was estimated with a pooled logistic regression model (eTable 4) for each system separately. The second weight was an inverse probability of remaining uncensored weight (IPCW, eTable 5). This is using the probability of remaining uncensored at each timepoint, conditional on remaining uncensored up to that point (cumulative probability). The mechanism of censoring was different by treatment group, for example unboosted residents can censor when they subsequently receive the vaccine but boosted residents do not. Pooled logistic regression models were used to estimate the IPCW for boosted and unboosted arms separately. The product of the weights was taken (IPW = IPTW * IPCW) and used in an outcome regression model. Weights were truncated at the 1% and 99% intervals. We fit a pooled logistic regression model of a dataset which was expanded for each discrete time interval (days) with dummy variables for each timepoint, f(t), and an interaction with treatment arm, weighted by IPW.

$$Logit[\Pr(Y_{t+1} \mid Y_t = 0, A)] = \beta_0 + \beta_1 f(t) + \beta_2 A + \beta_3 [A * f(t)]$$

An event-free survival probability at each timepoint and outcome was calculated from the model. Both cohorts used the same analytical approach, but statistical models differ because of underlying differences in the data and separate analysts performing model selection for the two nursing home systems (e.g. the Veteran population is mostly male, different age distribution).

© 2022 McConeghy KW et al. *JAMA Network Open*.

eFigure 1. Flowchart of Eligibility for Target Trials of mRNA Boosters in 2 US Nursing Home Systems

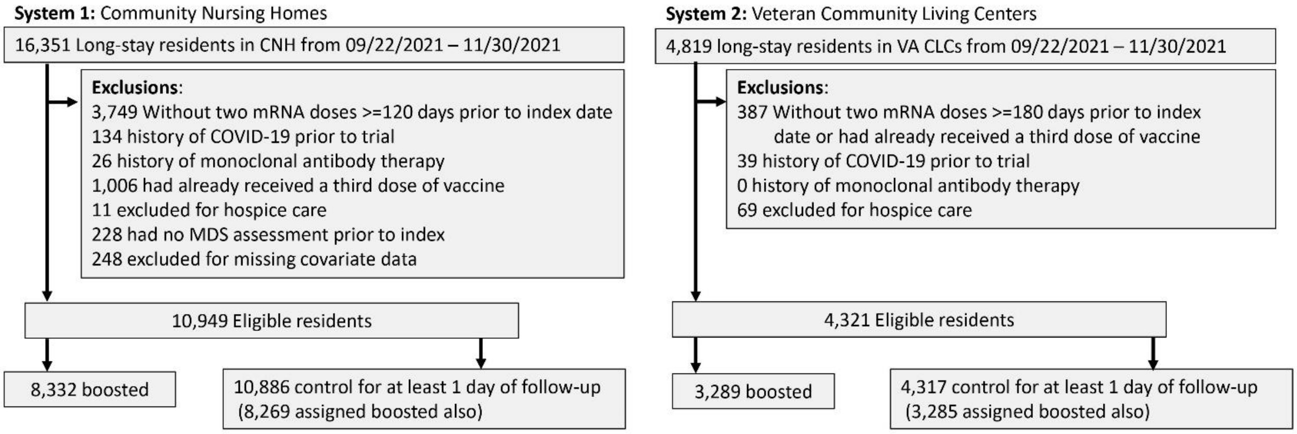

Description. Each system include residents present in the home, and meeting long-stay definitions (100 days in the home, with a gap of no more than 10 days). Further exclusion criteria are outlined.

© 2022 McConeghy KW et al. *JAMA Network Open*.

eFigure 2. Covariate Balance Before and After Weighting

System 1 – Community Nursing Homes

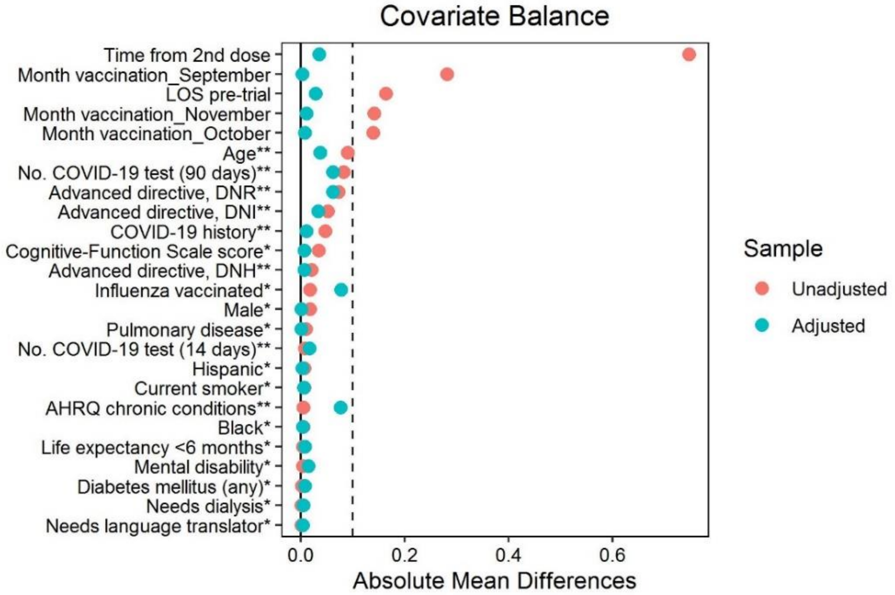

System 2 – Veteran Affairs, Community-Living Centers

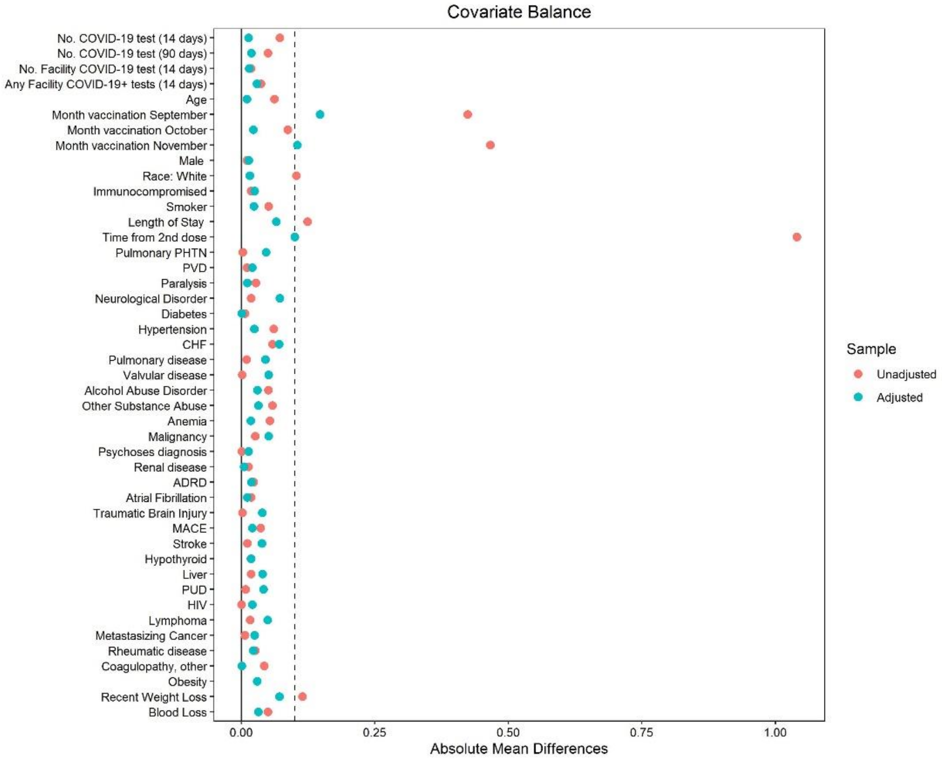

Description. The plot depicts the standardized mean differences (SMD) in boosted and non-boosted residents. Unadjusted refers to differences before applying probability weights. Adjusted is after applying inverse probability weighting. Variable definitions; LOS Pretrial – Length of stay before index date;

© 2022 McConeghy KW et al. *JAMA Network Open*.

Advanced directives DNR – do not resuscitate, DNI – do not intubate; DNH – do not hospitalize; HTN – hypertension, PVD – peripheral vascular disease, CHF – congestive heart failure, ADRD – Alzheimer's disease and related dementia, MACE – major adverse cardiovascular event (e.g. stroke, MI), PUD – peptic ulcer disease, HIV – human immunodeficiency virus. Items with an * are collected from nursing home minimum dataset 3.0, otherwise data is collected from the electronic health record and diagnoses classified according to Elixhauser ICD-10-CM comorbidity definitions. The x-intercept line is at 0.1 SMD is given to reference a reasonable maximally acceptable difference between groups.

© 2022 McConeghy KW et al. *JAMA Network Open*.

eTable 1. Definition of Immunocompromised Status

System 1 Immunocompromised definition	
One of any of the following in the 6 months prior to the index date was used to classify individuals as immunocompromised	
**Medications**	**Diagnosis codes**
5-fluorouracil, Abatacept, Adalimumab, Altretamine, Anakinra, Azathioprine Basiliximab, Belatacept, Bendamustine, Budesonide, Busulfan Carmustine, Certolizumab, Chlorambucil Cisplatin, Cyclophosphamide, Cyclosporine, Calcineurin, Cytarabine Diaziquone, Etanercept, Everolimus Golimumab, Hydrocortisone, Hydroxychloroquine, Ixekizumab Ifosfamide, Infliximab, Lomustine Mechlorethamine, Melphalan, Mercaptopurine, Methotrexate, Mycophenolate Natalizumab, Oxaliplatin, Procarbazine, Rituximab Secukinumab, Sirolimus, streptozocin Tacrolimus, temozolomide Thioguanine, thiotepa, Tocilizumab, Tofacitinib, Ustekinumab Vedolizumab	**Any code or branching code of the following:** Z51.11, Z51.12, Z51.0, Z99.2, Z49.3, M35.9, L93.0, Z94, Z49, B20, D61, D70, D71, D72, D73, D86, D89, C81, C82, C83, C84, C85, C86, C87, C88, C89, C90, C91, C92, C93, C94, C95, C96, D37, D38, D39, D40, D41, D42, D43, D44, D45, D46, D47, D48, M04, K50, K51, K52, M05, M06, M32, M34
**System 2 Immunocompromised status**	
Abatacept, Abemaciclib, Acalabrutinib, Adalimumab, Ado-trastuzumab, Afatinib, Aldesleukin, Alectinib, Alefacept, Alemtuzumab, Alpelisib, Altretamine, Amifostine, Anakinra, Anti-thymocyte globulin, Apremilast, Arsenic, Asparaginase, Atezolizumab, Atezolizumab, Auranofin, Avapritinib, Avelumab, Aurothioglucose , Axicabtagene, Axitinib , Azacitidine, Azathioprine , Baricitinib, Basiliximab, Belatacept, Belimumab, Belinostat, Belumosudil, Bendamustine, Bevacizuma, Bexarotene, Binimetinib , Bleomycin, Blinatumomab, Bortezomib, Bosutinib , Brentuximab, Brigatinib , Brodalumab, Busulfan, Cabazitaxel, Cabozantinib, Calaspargase, Canakinumab, Capecitabine, Carboplatin, Carfilzomib, Carmustine, Cemiplimab, Ceritinib, Certolizumab, Cetuximab, Chlorambucil, Cisplatin, Cladribine, Clofarabine, Cobimetinib, Copanlisib, Crizotinib, Cyclophosphamide,	**Any code or branching code of the following:** Z51.11, Z51.12, Z51.0, Z94, Z99.2, Z49, B20, D91, D70, D71, D72, D73, D75.81, D86, D89, C81, C82, C83, C84, C85, C86, C87, C88, C89, C90, C91, C92, C93, C94, C95, C96, D37, D38, D39, D40, D41, D42, D43, D44, D45, D46, D47, D48, M04, M05, M06, K50, K51, K52, M35.9, M32, L93.0, M34

© 2022 McConeghy KW et al. *JAMA Network Open*.

Cyclosporine, Cytarabine, Dabrafenib, Dacarbazine, Daclizumab, Dacomitinib, Dactinomycin, Daratumumab, Dasatinib, Daunorubicin, Decitabine, Denileukin, Denosumab, Dimethyl fumarate, Dinutuximab, Diroximel fumarate, Docetaxel, Doxorubicin, Durvalumab, Eculizumab, Efalizumab, Elotuzumab, Emapalumab, Enasidenib, Encorafenib, Entrectinib, Enzalutamide, Epirubicin, Erdafitinib, Eribulin, Erlotinib, Estramustine, Etanercept, Etoposide, Everolimus, Fedratinib, Fingolimod, Floxuridine, Fludarabine, Fluorouracil, Flutamide, Fostamatinib, Fremanzumab, Gefitinib, Gemcitabine, Gemtuzumab, Gilteritinib, Glasdegib, Glatiramer, Glucarpidase, Golimumab, Guselkumab, Hydroxyurea, Ibritumomab, Ibrutinib, Idarubicin, Idelalisib, Ifosfamide, Imatinib, Infliximab, Inotuzumab, Inotuzumab, Interferon beta-1a, Interferon beta-1b, Ipilimumab, Irinotecan, Ivosidenib, Ixabepilone, Ixazomib, Ixekizumab, Lanadelumab, Lapatinib, Larotrectinib , Leflunomide, Lenalidomide , Lenvatinib, Lomustine, Lorlatinib, Lurbinectibdin, Mechlorethamine, Melphalan, Mercaptopurine, Methotrexate, Mitomycin , Mitotane, Mitoxantrone, Mogamulizumab, Mmuromonab, Mycophenolic acid, Natalizumab, Mycophenolate mofetil, Necitumumab, Nelarabine, Nilotinib, Nilutamide, Niraparib , Nivolumab, Obinutuzumab, Ocrelizumab, Ofatumumab, Olaparib, Olaratumab, Omacetaxine, Omalizumab , Osimertinib, Oxaliplatin, Paclitaxel, Palbociclib, Palivizumab, Panitumumab , Panobinostat, Pazopanib, Pegademase, Pegaspargase, Peginterferon/ribavirin, Peginterferon, Pembrolizumab, Pemetrexed, Pentostatin, Pertuzumab, Pexidartinib, Plicamycin, Pipobroman, Polatuzumab, Pomalidomide, Ponatinib, Pralatrexate, Procarbazine, Ramucirumab, Rasburicase, Ravulizumab, Regorafenib, Ribociclib, Rilonacept, Risankizumab, Rituximab, Romosozumab, Romidepsin, Rucaparib, Ruxolitinib, Sarilumab, Secukinumab, Selinex, Siltuximab, Siponimod, Sipuleucel, Sirolimus, vinblastine, Sonidegib, Sorafenib,	

Streptozocin, Sunitinib, Tacrolimus, Talazoparib, Talimogene, Tazemetostat , Temozolomide, Temsirolimus, Teniposide, Teriflunomide, Thalidomide, Thioguanine, Thiotepa, Tildrakizumab, Tildrakizumab, Tocilizumab, Tofacitinib, Topotecan, Tositumomab, Trabectedin, Trametinib , Trastuzumab, Trifluridine, Upadacitinib, Uracil mustard, Ustekinumab, Valrubicin, Vandetanib, Vedolizumab, Vemurafenib, Venetoclax, Vinblastine, Vincristine , Vinorelbine, Vismodegib , Voclosporin, Zanubrutinib	
**Description.** The list of medications and diagnosis codes which were used to identify immunocompromised residents. System 2 includes a more comprehensive list developed and validated internally by Pharmacy Benefits Management for operational or research use in a variety of applications. It includes many medications that may be less relevant to a nursing home population, but is used for internal consistency in measurement of Veteran health outcomes across studies.	

© 2022 McConeghy KW et al. *JAMA Network Open*.

eTable 2. System 1 Trial Eligibility by Date

Trial Date	Present in home for 100 days	No discharge plan	Primary mRNA vaccination completed >=120 days prior	No history of SARS-CoV-2 in past 90 days	No history of monoclonal antibody use in past 90 days	Had not already received booster dose	Not currently receiving hospice care
9/22/2021	14417	14417	10868	10585	10550	10013	10003
9/23/2021	14435	14435	10924	10638	10603	10018	10008
9/24/2021	14472	14472	10955	10667	10632	10018	10008
9/27/2021	14538	14538	10984	10692	10657	10034	10024
9/28/2021	14525	14525	10973	10684	10647	9995	9985
9/29/2021	14537	14537	10994	10706	10669	10010	10000
9/30/2021	14551	14551	11008	10715	10679	9940	9930
10/1/2021	14555	14555	11017	10722	10680	9765	9755
10/4/2021	14617	14617	11057	10738	10699	9730	9720
10/5/2021	14594	14594	11046	10725	10686	9547	9537
10/6/2021	14599	14599	11081	10754	10714	9441	9431
10/7/2021	14619	14619	11097	10763	10723	9187	9179
10/8/2021	14632	14632	11124	10790	10748	8812	8804
10/11/2021	14691	14691	11157	10811	10769	8605	8597
10/12/2021	14679	14679	11156	10809	10765	8437	8429
10/13/2021	14662	14662	11158	10809	10765	8235	8228
10/14/2021	14667	14667	11173	10814	10768	7698	7693
10/15/2021	14674	14674	11182	10821	10776	7439	7434
10/18/2021	14734	14734	11233	10865	10818	7284	7279
10/19/2021	14715	14715	11221	10862	10813	7143	7138
10/20/2021	14718	14718	11243	10885	10837	6715	6711
10/21/2021	14731	14731	11252	10894	10846	6474	6469
10/22/2021	14742	14742	11277	10915	10868	6216	6209
10/25/2021	14776	14776	11289	10933	10887	6103	6096
10/26/2021	14759	14759	11282	10921	10876	6034	6027
10/27/2021	14755	14755	11298	10938	10894	5931	5926
10/28/2021	14767	14767	11298	10943	10895	5713	5708
10/29/2021	14783	14783	11320	10960	10910	5502	5497
11/1/2021	14805	14805	11326	10951	10900	5116	5112
11/2/2021	14781	14781	11305	10927	10876	5012	5008
11/3/2021	14785	14785	11318	10935	10884	4788	4784
11/4/2021	14788	14788	11341	10945	10894	4447	4443
11/5/2021	14784	14784	11344	10953	10902	4002	3998
11/8/2021	14786	14786	11347	10952	10904	3537	3533
11/9/2021	14770	14770	11341	10949	10901	3425	3421

© 2022 McConeghy KW et al. *JAMA Network Open*.

11/10/2021	14762	14762	11344	10952	10905	3340	3336
11/11/2021	14769	14769	11364	10972	10925	3199	3195
11/12/2021	14783	14783	11390	11004	10956	2962	2958
11/15/2021	14803	14803	11401	11030	10977	2822	2818
11/16/2021	14782	14782	11393	11021	10971	2787	2783
11/17/2021	14785	14785	11405	11037	10986	2691	2687
11/18/2021	14789	14789	11442	11028	10987	2610	2606
11/19/2021	14787	14787	11450	11033	10991	2500	2496
11/22/2021	14801	14801	11454	11050	11008	2359	2355
11/23/2021	14770	14770	11437	11044	11003	2270	2266
11/24/2021	14749	14749	11429	11036	10997	2087	2083
11/25/2021	14751	14751	11437	11050	11012	2050	2046
11/26/2021	14747	14747	11443	11058	11019	2060	2056
11/29/2021	14745	14745	11435	11065	11024	2066	2062
11/30/2021	14725	14725	11422	11058	11014	2039	2035

© 2022 McConeghy KW et al. *JAMA Network Open*.

eTable 3. System 2 Trial Eligibility by Date

Date	Present in nursing home	Long-term resident	Primary mRNA Vaccination Completed >= 180 days prior & not yet boosted	No History of SARS-COV-2 in past 90 days	No History of monoclonal antibody use in past 90 days	Not currently receiving hospice care
9/22/2021	6444	4506	4187	4024	4021	3861
9/23/2021	6440	4495	4180	4018	4015	3854
9/24/2021	6434	4487	4173	4012	4009	3849
9/25/2021	6368	4479	4167	4008	3999	3839
9/26/2021	6357	4481	4170	4013	4003	3844
9/27/2021	6416	4492	4167	4010	4000	3846
9/28/2021	6469	4498	4156	3996	3986	3838
9/29/2021	6466	4498	4047	3892	3882	3736
9/30/2021	6472	4499	3879	3739	3729	3589
10/1/2021	6461	4484	3729	3590	3580	3449
10/2/2021	6388	4473	3598	3469	3459	3332
10/3/2021	6365	4467	3593	3465	3455	3331
10/4/2021	6396	4470	3595	3468	3458	3334
10/5/2021	6426	4479	3532	3405	3395	3276
10/6/2021	6439	4487	3447	3318	3308	3195
10/7/2021	6449	4494	3243	3124	3114	3007
10/8/2021	6468	4482	3150	3036	3026	2921
10/9/2021	6392	4473	3102	2988	2978	2875
10/10/2021	6375	4479	3106	2992	2982	2877
10/11/2021	6368	4485	3111	2997	2987	2882
10/12/2021	6423	4500	3124	3010	3000	2891
10/13/2021	6431	4507	3122	3007	2997	2891
10/14/2021	6455	4513	3115	2998	2988	2882
10/15/2021	6463	4503	3071	2954	2944	2843
10/16/2021	6385	4497	3040	2923	2913	2809
10/17/2021	6357	4503	3044	2927	2916	2813
10/18/2021	6405	4504	3044	2918	2907	2806
10/19/2021	6417	4507	2999	2874	2863	2763
10/20/2021	6409	4515	2955	2830	2819	2719
10/21/2021	6402	4520	2939	2815	2804	2702
10/22/2021	6410	4510	2906	2787	2776	2675
10/23/2021	6330	4498	2874	2755	2744	2645
10/24/2021	6312	4504	2879	2758	2747	2648
10/25/2021	6367	4519	2892	2768	2757	2659
10/26/2021	6370	4514	2886	2763	2752	2653
10/27/2021	6378	4516	2794	2673	2662	2564

10/28/2021	6405	4516	2618	2501	2490	2398
10/29/2021	6429	4502	2423	2307	2296	2208
10/30/2021	6362	4494	2248	2135	2124	2044
10/31/2021	6349	4497	2251	2137	2126	2047
11/1/2021	6411	4503	2252	2137	2126	2050
11/2/2021	6436	4506	2150	2037	2026	1958
11/3/2021	6434	4509	2036	1926	1915	1850
11/4/2021	6452	4499	1885	1777	1766	1705
11/5/2021	6456	4482	1811	1705	1694	1634
11/6/2021	6373	4472	1733	1631	1620	1559
11/7/2021	6357	4478	1735	1635	1625	1565
11/8/2021	6414	4489	1741	1647	1638	1580
11/9/2021	6443	4498	1650	1557	1548	1492
11/10/2021	6455	4498	1595	1507	1498	1446
11/11/2021	6382	4497	1433	1347	1338	1288
11/12/2021	6433	4492	1422	1337	1328	1278
11/13/2021	6371	4486	1354	1270	1261	1209
11/14/2021	6350	4491	1347	1264	1255	1203
11/15/2021	6401	4500	1352	1269	1260	1208
11/16/2021	6434	4508	1275	1195	1187	1136
11/17/2021	6447	4513	1130	1047	1038	990
11/18/2021	6462	4510	1074	992	983	936
11/19/2021	6451	4499	988	910	901	855
11/20/2021	6382	4491	937	859	850	803
11/21/2021	6359	4487	938	861	852	805
11/22/2021	6385	4484	938	861	852	807
11/23/2021	6393	4481	906	834	825	781
11/24/2021	6373	4476	873	802	792	750
11/25/2021	6291	4483	853	785	775	733
11/26/2021	6316	4479	854	787	777	735
11/27/2021	6272	4466	838	770	760	718
11/28/2021	6252	4471	839	774	763	721
11/29/2021	6309	4478	842	775	764	722
11/30/2021	6352	4478	829	762	751	711

© 2022 McConeghy KW et al. *JAMA Network Open*.

eFigure 3. Relative Booster Effectiveness in First 12 Weeks Among Nursing Home Residents

System 1

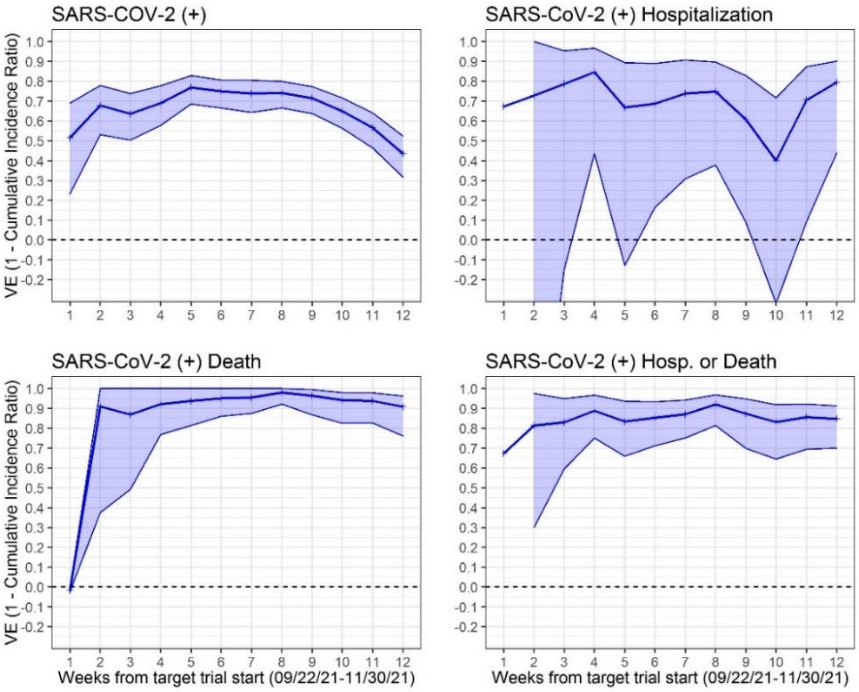

System 2

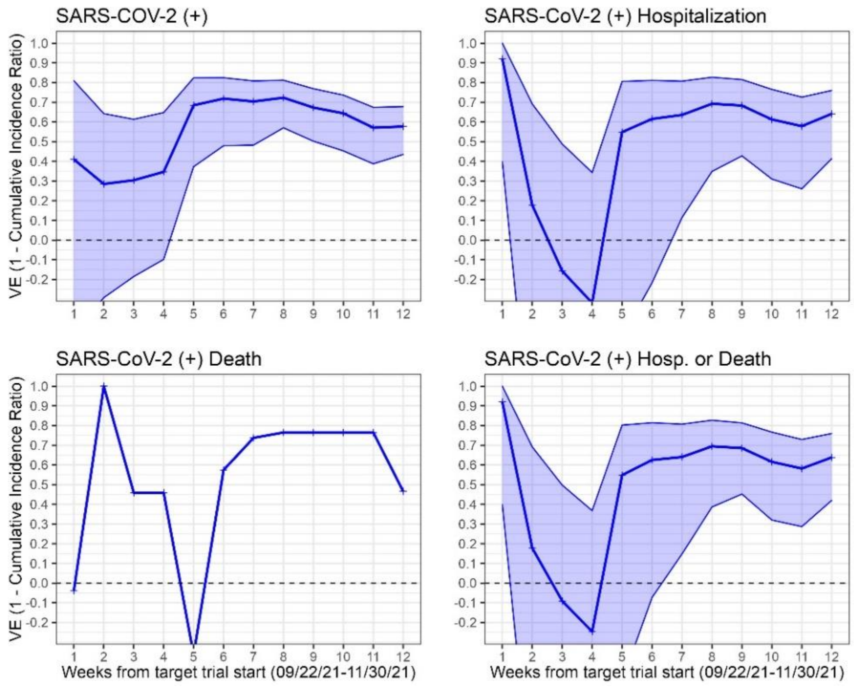

Description. Each panel represents the vaccine effectiveness as 1 – the cumulative incidence rate ratios for each outcome. Shaded regions represent 95% confidence intervals. The confidence interval is omitted for certain timepoints and panels due to instability and difficulty in estimation. The point estimates at week 12 are reported in Table 3.

© 2022 McConeghy KW et al. *JAMA Network Open*.

eTable 4. Coefficients From Model Estimating Inverse Probability of Treatment Weights

	**System 1 Community Nursing Homes**		**System 2 VA Community Living Centers**	
	**Estimate**	**Std. Error**	**Estimate**	**Std. Error**
(Intercept)	-4.648	0.203	-0.827	1.868
COVID-19 tests, prior 90 days*	0.001	0.002	1.245	0.383
COVID-19 tests, prior 90 days**	-	-	2.555	0.638
COVID-19 tests, prior 90 days***	-	-	2.289	0.458
October date versus September	1.981	0.061	-2.346	2.532
November date versus September	3.060	0.075	-19.947	5.128
Facility tests for COVID-19 in prior 2 weeks	-	-	-0.002	0.001
Facility COVID outbreak in prior 2 weeks	-	-	0.686	0.142
Age at index	0.001	0.001	0.002	0.003
Male versus Female	0.010	0.036	-0.072	0.152
race white versus other	0.000	0.045	-0.133	0.061
Immunocompromised	0.248	0.055	0.075	0.066
Smoker	-	-	0.152	0.092
length of stay prior to index	-	-	0.000	0.000
Time from second dose*	2.325	0.089	4.075	1.051
Time from second dose**	3.696	0.306	3.223	4.454
Time from second dose***	1.415	0.173	20.803	3.353
Prior hospitalization in 90 days	-0.164	0.051	-	-
Fixed effects for state	Included	-	Not included	-
I(COVID-19 tests*, October)	-	-	-1.933	0.455
I(COVID-19 tests**, October)	-	-	-3.866	0.718
I(COVID-19 tests***, October)	-	-	-3.290	0.551
I(COVID-19 tests*, November)	-	-	-2.040	0.485
I(COVID-19 tests**, November)	-	-	-2.012	0.761
I(COVID-19 tests ***, November)	-	-	-2.412	0.582
I(Facility testing rate, October)	-	-	0.003	0.001
I(Facility testing rate, November)	-	-	0.004	0.001
I(Facility COVID outbreak, October)	-	-	-0.517	0.172
I(Facility COVID outbreak, November)	-	-	-0.717	0.200
I(Time from second dose*, October)	-	-	-0.234	1.383
I(Time from second dose*, November)	-	-	8.890	2.751
I(Time from second dose**, October)	-	-	7.186	5.766
I(Time from second dose**, November)	-	-	38.886	10.598
I(Time from second dose***, October)	-	-	-4.752	3.638
I(Time from second dose***, November)	-	-	-4.711	4.139

Description. Models where the dependent variable was receipt of booster dose on index date. Fixed effects for states were not included in System 2, because most states only have one facility. The logistic regression models were estimated separately in the two datasets by different analysts but followed similar principles for model selection. *,**,*** refer to spline basis terms, a '-' means that term was not included in the model. I=Interaction term.

© 2022 McConeghy KW et al. *JAMA Network Open*.

eTable 5. Coefficients From Model Estimating Inverse Probability of Censoring Weights

	System 1[a]				System 2[b]			
	Boosted arm		Unboosted arm		Boosted arm		Unboosted arm	
	Estimate	Std. Error	Estimate	Std. Error	Estimate	Std. Error	Estimate	Std. Error
(Intercept)	11.019	0.382	4.058	0.176	19.611	1022	15.230	292
Age at index	0.003	0.001	-0.001	0.001	-0.010	0.013	-	-
Immunocompromised status			-0.068	0.033	-0.373	0.271	-0.207	0.088
Length of stay*	0.351	0.119	-	-	7.337	3.948	-0.215	0.338
Length of stay**	0.985	0.257	-	-	-0.587	9.020	2.599	0.550
Time from second dose*	0.305	0.210	-1.400	0.133	2.490	1.303	0.025	0.772
Time from second dose**	-9.804	0.758	-1.191	0.452	0.928	1.299	4.973	4.038
Length of stay***	0.274	0.473	-	-	-	-	2.197	0.972
Time from second dose***	-2.450	0.172	-1.201	0.542	-	-	-4.804	4.088
I(Time from second dose*, October)	-	-	0.610	0.156	-	-	-1.756	0.933
I(Time from second dose*, November)	-	-	1.379	0.169	-	-	-4.285	1.377
I(Time from second dose**, October)	-	-	-1.673	0.542	-	-	-7.164	4.641
I(Time from second dose**, November)	-	-	-3.099	0.575	-	-	-12.544	6.629
I(Time from second dose***, October)	-	-	0.567	0.602	-	-	-0.881	3.842
I(Time from second dose***, November)	-	-	0.894	0.578	-	-	5.056	4.451
Male versus Female	-0.028	0.028	0.022	0.022	-	-	-0.023	0.222
Facility tests for COVID-19 in prior 2 weeks	-	-	0.019	0.006	-	-	0.052	0.030
Facility COVID outbreak in prior 2 weeks	-	-	-	-	-	-	-0.001	0.000
October date versus September	-1.354	0.073	0.754	0.204	-	-	4.215	1.898
November date versus September	-2.973	0.084	1.322	0.219	-	-	8.986	2.877
Black versus other	-	-	-	-	-	-	0.014	0.094
Smoker	-	-	-	-	-	-	-0.157	0.124
Prior hospitalization in past 90 days	-0.402	0.041	-0.148	0.030	-	-	-	-
Do not resuscitate order	-	-	-0.097	0.021	-	-	-	-

Description. Models where the dependent variable was not censoring at a follow-up timepoint in a pooled regression model of person-time observations. The data for System 1 was expanded in time units of days, while in System 2, units of weeks (this makes the scale of coefficients different in either model, so values are not directly comparable). The logistic regression models were estimated separately in the two datasets by different analysts who followed similar principles in model selection. *,**,*** refer to spline basis terms, a '-' means that term was not included in the model. I=Interaction term. The time coefficients are excluded from the output for brevity. [a]In System 1, a restricted cubic spline with 4 degrees of freedom was used for time, [b]System 2 used fixed effects for each timepoint.

© 2022 McConeghy KW et al. *JAMA Network Open*.
